# Minocycline-Associated Pseudotumor Cerebri with Severe Papilledema

**DOI:** 10.1155/2009/203583

**Published:** 2010-02-16

**Authors:** Simon R. Bababeygy, Michael X. Repka, Prem S. Subramanian

**Affiliations:** ^1^Department of Ophthalmology, The Johns Hopkins Hospitals, 600 N. Wolfe St, Baltimore, MD 21287, USA; ^2^Howard Hughes Medical Institute, Stanford University School of Medicine, Stanford, CA 94305, USA; ^3^Wilmer Eye Institute, Maumenee 127, 600 N. Wolfe Street, Baltimore, MD 21287, USA

## Abstract

*Background*. Pseudotumor cerebri is an acknowledged but unusual complication of oral minocycline use. Vision loss and papilledema have been described as mild and transient, and some authors suggest that treatment is not needed. *Methods*. Case series of 2 patients with severe papilledema and visual field loss. *Results*. Severe pseudotumor cerebri developed in 2 nonobese patients taking minocycline. Their disease required further treatment even upon drug discontinuation because of visual field loss and papilledema. *Conclusions*. Minocycline-associated pseudotumor cerebri is not always a self-limited condition and may require aggressive medical or surgical management.

## 1. Introduction

The pathogenesis of pseudotumor cerebri syndrome (PTC) is poorly understood, although its diagnostic criteria are well established [1]. While most cases are idiopathic, recent data indicate that patients with severe forms of PTC may have partial venous outflow obstruction [2–4], and such patients may have greater long-term morbidity. Antibiotic medications such as minocycline, tetracycline, and doxycycline have also been repeatedly implicated as a causative or contributory factor in PTC [5–7]. The prognosis of minocycline-related PTC reported in the literature is quite variable. Some authors suggest it is a benign condition that resolves spontaneously upon discontinuing the antibiotic [8, 9], while others report permanent vision loss may ensue [5]. It is possible that venous sinus thrombosis was a confounding factor regarding disease severity in some instances, as most reported cases predate reliable magnetic resonance venography (MRV). Here we describe two cases of minocycline-induced PTC with severe papilledema and normal MRV, indicating that minocycline alone may be associated with severe PTC without venous sinus anomalies.

## 2. Materials and Methods

Retrospective case analysis was performed on cases of pseudotumor cerebri seen by two of the authors (Michael X.Repka, Prem S. Subramanian) in their clinical practices. A specific search for nonobese patients (BMI ≤ 25) with minocycline use at the time of pseudotumor cerebri diagnosis was made, and cases identified are presented below. IRB approval was not required for this study by our institutional guidelines, since it involves a case series of fewer than 3 patients. 

## 3. Results

### 3.1. Case  1

**Figure 1:**
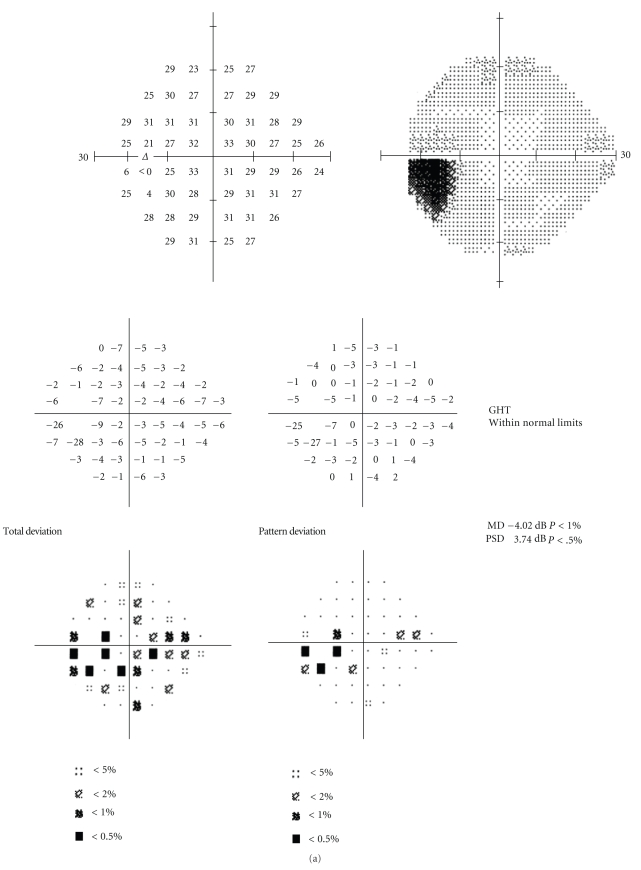

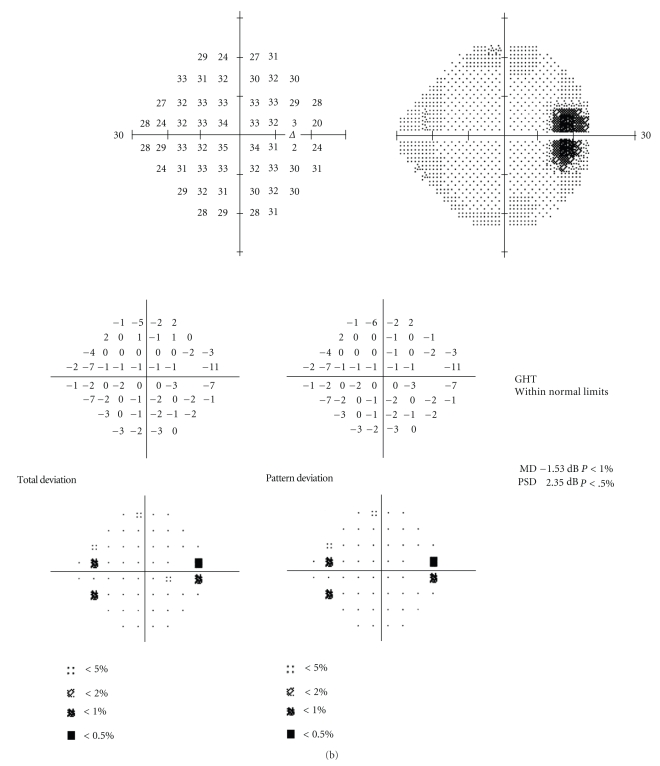
Automated perimetry results, case 1. In the left eye (a), the mean deviation was −4.02 with blind spot enlargement as well as some depression extending toward fixation from the blind spot. In the right eye (b), there was a mean deviation of −1.53 and slight enlargement of the blind spot.

A 23-year-old woman with a 4-month history of doxycycline treatment and a subsequent 3-month history of minocycline treatment for acne developed tinnitus, vomiting, phonophobia, and photophobia along with severe headaches and progressive horizontal binocular diplopia. She was otherwise healthy and took no other medications.Visual acuity was 20/15 in the right eye and 20/20 in the left eye with a left relative afferent pupillary defect.Humphrey visual fields revealed a triangular scotoma in her left eye (Figure 1). 

Severe bilateral papilledema was present, greater on the left than the right. Lumbar puncture demonstrated an opening pressure of 41 cm H_2_O with normal cerebrospinal fluid. Minocycline use was discontinued. Head CT and contrast-enhanced MRI were both normal and an MRV showed no venous thrombosis or stasis. The patient's symptoms of diplopia improved 3 weeks later, but papilledema and headache persisted with worsening of visual fields. Repeat MRV was normal. The intracranial pressure remained elevated at 33 cm H_2_O despite treatment with 2 g acetazolamide/day. Ventriculoperitoneal shunting was performed for intracranial pressure control.

### 3.2. Case  2

**Figure 2:**
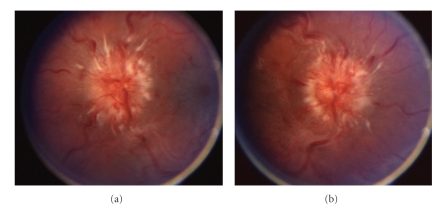
Optic disc appearance, case 2. Fundoscopy of the left eye (a) and right eye (b) reveals grade IV papilledema as evidenced by severe elevation and hemorrhages.

A 15-year-old girl with a 6-week history of minocycline treatment for acne vulgaris developed progressive bifrontal headache with pain around the right eye, vertical binocular diplopia, nausea, neck pain, and muscle spasm. Visual acuity was 20/20-2 in the right eye and 20/15-1 in the left eye. Threshold visual fields revealed enlarged blind spots in both eyes, but otherwise normal findings. Dilated ophthalmoscopy disclosed florid papilledema in both eyes with peripapillary hemorrhages and choroidal folds (Figure 2). Head CT and enhanced MRI were both normal, and an MRV showed no evidence of venous thrombosis. The opening pressure was 55 cm H_2_O  with normal CSF formula. Minocycline was discontinued. Acetazolamide treatment was prescribed, then discontinued 5 months later after resolution of the papilledema, headaches, visual field defects, and diplopia.

## 4. Discussion

We present 2 cases of minocycline-associated PTC in nonobese patients in whom symptoms were severe, though there were no signs of venous sinus thrombosis on MRV. In one case, papilledema and elevated ICP persisted for more than 3 months despite stopping minocycline. Vision loss ensued in one patient despite maximal medical therapy. Duration of PTC can never be known for sure, and it is possible that both patients had disc swelling for some time before diagnosis and thus suffered vision loss as a result. However, vision loss and papilledema in PTC found incidentally are usually mild. There is conflicting evidence in the literature regarding the prognosis of minocycline-induced PTC. In the largest published series of minocycline-treated patients, consisting of 12 patients, 6 had severe papilledema at diagnosis and 3 had residual visual field loss [5]. Kesler et al. reported 18 patients with a history of tetracycline use associated with pseudotumor cerebri and found that 6 patients had a relapsing course, while 12 patients had rapid recovery from the disease upon stopping the antibiotic agent [10]. The authors suggested that tetracycline played only a minor role in the patients who relapsed; however, a number of their patients were obese [10], in contrast to the patients we present in this report.

 Others have stated that minocycline-induced PTC usually has minimal visual consequence and resolves rapidly upon discontinuing the medication [8]. Normalization of intracranial pressure within 1 month of stopping tetracycline has been reported as well [9]. However, the mechanism by which tetracycline or minocycline may induce pseudotumor cerebri has not been determined. Our patient in case 1 was treated with both doxycycline and then minocycline, although her symptoms did not arise until the latter drug was used. Our findings suggest that minocycline alone may induce severe PTC with persistently elevated intracranial pressure, and patients with this condition may require medical and surgical treatment beyond discontinuation of the medication. We propose that minocycline-associated pseudotumor cerebri is not a benign condition and must be followed closely with aggressive interventions to prevent vision loss.
